# Hybrid Thermodynamic and Machine Learning Optimization of Plate Heat Exchanger Performance for Brewery Cooling Systems in Uganda

**DOI:** 10.12688/openresafrica.16068.1

**Published:** 2025-11-03

**Authors:** Byamanywoha Edgar, Val Hyginus Udoka Eze, Saadelnour Abdueljabbar Adam, Sanusi Yinka, Bubu Pius Erheyovwe

**Affiliations:** 1 Department of Electrical, Telecom. and Computer Engineering, Kampala International University - Western Campus, Bushenyi, Western Region, 2000, Uganda; 2 Department of Mechanical Engineering, Kampala International University - Western Campus, Bushenyi, Western Region, 2000, Uganda

**Keywords:** Plate Heat Exchanger, Cooling Optimization, Artificial Neural Networks, Thermal Efficiency, Fouling Analysis

## Abstract

**Background:**

Efficient thermal management in brewing is critical for product quality, energy efficiency, and sustainability. At Nile Breweries Limited, Mbarara, Uganda, the Plate Heat Exchanger (PHE) cooling system has faced persistent challenges in achieving the target wort outlet temperature of 283 K (10 °C) or lower. Understanding and optimizing the thermodynamic and operational performance of this system is essential, particularly in resource-constrained Sub-Saharan production environments.

**Methods:**

An integrated optimization framework was developed by combining thermodynamic modeling, empirical diagnostics, and machine learning. Over 50 production batches were monitored using a high-resolution (1 Hz) dataset to assess short-term performance, while a longer dataset of 480 batches was analyzed for fouling trends. Statistical regression, LMTD analysis, and correlation tests were applied to evaluate thermal relationships, and a feedforward Artificial Neural Network (ANN) was trained on seven operational parameters in MATLAB/Simulink to enable predictive control.

**Results:**

Only 14.60% of production batches achieved the cooling target (95% CI: 11.2%–18.7%). Regression analysis showed a significant positive association between wort mass flow rate and outlet temperature (slope = 1.24 K/(kg/s), p < 0.001, R ^2^ = 0.42), and a negative correlation with heat exchanger effectiveness (slope = –0.0037 per kg/s, p = 0.012, adjusted R ^2^ = 0.28). LMTD decreased with increasing wort flow (slope = –0.42 K/(kg/s), p = 0.002, R ^2^ = 0.36), revealing a throughput–efficiency trade-off. Water flow rate improvements diminished beyond 12.1 kg/s, showing no significant effect on effectiveness (p = 0.18) or LMTD (p = 0.15). Fouling analysis indicated a gradual decline in mass-corrected effectiveness across 480 batches (slope = –0.00001 per batch, p < 0.001, R ^2^ = 0.22), while wort pH exerted a minor negative effect on effectiveness (r = –0.1645). The ANN demonstrated strong predictive accuracy (R ^2^ = 0.8762, MSE = 0.0578, MAE = 0.0938), with residuals confirming minimal bias. Optimization identified optimal operating ranges of 16–17 kg/s for water flow and <16.5 kg/s for wort flow.

**Conclusions:**

The study highlights a significant performance shortfall in the brewery’s cooling system, driven primarily by throughput–efficiency trade-offs, diminishing thermal returns, and progressive fouling. By integrating classical thermodynamic analysis with machine learning, the research demonstrates a robust and scalable framework for enhancing energy efficiency and adaptive process control in brewing and other energy-intensive industries. This work advances the application of intelligent thermal management strategies in Sub-Saharan industrial contexts, with future research recommended in real-time fouling detection, economic optimization of flow control, and hybrid predictive modeling for dynamic production environments.

## Introduction

In modern industrial systems, heat exchangers serve as critical thermal management devices designed to facilitate efficient energy transfer between fluid streams while maintaining strict physical separation to prevent cross-contamination ( Klemeš *et al.*, 2021; Mahmoudinezhad *et al.*, 2023). Among the various configurations, Plate Heat Exchangers (PHEs) have emerged as a preferred solution due to their high thermal efficiency, compact design, and adaptability to a wide range of process conditions. Compared to conventional shell-and-tube heat exchangers, PHEs provide up to two to three times higher surface area-to-volume ratios, making them particularly effective in space-constrained applications where optimal heat transfer performance is essential ( Togun *et al.*, 2024; Yan & Yan, 2024). PHEs consist of a series of thin, corrugated metal plates arranged to form alternating flow channels for hot and cold fluids. This configuration promotes turbulent flow even at relatively low Reynolds numbers, thereby significantly enhancing convective heat transfer rates with minimal pressure drop penalties ( Abidin *et al.*, 2021; Chen *et al.*, 2024). Furthermore, their modular construction allows for ease of cleaning, maintenance, and future expansion. As such, PHEs have become widely adopted in diverse sectors including the chemical process industry, food and beverage manufacturing, pharmaceutical production, HVAC systems, and thermal energy recovery applications ( Carvajal-Mariscal *et al.*, 2022; Eze, 2025a; Eze *et al.*, 2025; Eze *et al.*, 2024; Eze *et al.*, 2024a; Eze *et al.*, 2024b; Shopska *et al.*, 2022).

In the brewing industry, PHEs play a vital role during the wort cooling stage, where the hot malt extract (wort) must be rapidly cooled from near-boiling temperatures (~95°C) to fermentation-ready conditions (~10°C or 283 K). This stage is critically important, as the rate and uniformity of cooling directly influence yeast viability, fermentation kinetics, microbial stability, and ultimately, the quality and consistency of the final beer product ( Jagiełło & Ludwig, 2024; Reis, 2023). Despite their benefits, the operational efficiency of PHEs can degrade over time due to fouling, scaling, corrosion, and uneven fluid distribution. These phenomena reduce the overall heat transfer coefficient (U), elevate pressure drops, and increase energy consumption ( Lin *et al.*, 2020; Lodhi & Agarwal, 2024). Evaluating the performance of PHEs requires a multifaceted approach involving thermal-hydraulic metrics such as Reynolds number, Nusselt number, fouling factor, and log mean temperature difference (LMTD), which collectively describe the system's efficiency and potential losses ( Jafari & Akyüz, 2022; Jiang *et al.*, 2020). In many industrial facilities, particularly in resource-limited settings, the lack of real-time monitoring systems and advanced diagnostics hinders timely intervention. This results in prolonged downtime, excessive utility consumption, and inconsistent product quality ( Sohrabi *et al.*, 2024; Watanabe *et al.*, 2024). These inefficiencies not only impact operational profitability but also constrain industries from achieving energy sustainability and environmental compliance targets ( Alfa, 2019; Toapanta-Ramos *et al.*, 2018).

Nile Breweries Limited (NBL), a major beer producer based in Mbarara, Uganda, and a subsidiary of AB InBev, relies on a plate heat exchanger system for wort cooling. The system is designed to reduce the wort temperature from ~95°C to the fermentation threshold of 10°C. However, operational data indicate frequent underperformance, with the outlet temperature consistently exceeding the required 283 K. This discrepancy leads to suboptimal fermentation conditions, elevated utility consumption, thermal losses, and increased carbon emissions. The resultant issues affect not only production efficiency but also flavor stability, product safety, and environmental performance. Addressing these deficiencies requires a comprehensive optimization strategy, encompassing heat exchanger geometry, plate material properties, turbulence promotion methods, flow arrangements, and control strategies. Although numerous studies have explored the influence of plate corrugation patterns and turbulence enhancement on PHEs ( Saleh & Sundar, 2021; Sandrin *et al.*, 2024), there exists a significant knowledge gap regarding context-specific optimization for breweries operating under tropical, resource-constrained conditions ( Albets-Chico *et al.*, 2013; Ali *et al.*, 2017; Aminian *et al.*, 2020; Awad *et al.*, 2013; Barletta *et al.*, 2013; Belyaev *et al.*, 2017; Bo-Fu *et al.*, 2012; Brian, 2004; Brian, 2008; Buongiorno, 2006).

This study seeks to fill this gap by investigating the technical and economic optimization of the PHE system at Nile Breweries, with the goal of achieving enhanced thermal performance, reduced fouling tendencies, and improved energy and water efficiency. The study’s focus aligns with global sustainability imperatives, particularly Sustainable Development Goal (SDG) 7 on affordable and clean energy and SDG 12 on responsible consumption and production. From an academic perspective, this work contributes to the field of applied thermal-fluid engineering by modeling, simulating, and validating the performance of an industrial-scale PHE under dynamic operating conditions. From a practical standpoint, it provides brewery operators with actionable insights to improve system efficiency, reduce environmental impacts, and extend equipment lifespan. The methodology employed integrates computational fluid dynamics (CFD) simulations, parametric optimization, and experimental validation, with specific emphasis on geometric and material variables influencing heat transfer behavior. By doing so, the study not only identifies performance bottlenecks but also proposes cost-effective retrofitting and redesign strategies suitable for deployment in comparable settings ( Cengel, 1985; Chandrasekhar, 1961; Chang, 2014; Edelweiss, 2020; Elsasser, 1955; Esfahani & Feshalami, 2018; Eze, 2025; Faber, 1995; Gholinia *et al.*, 2018; Global Data Energy, 2017; Hamzah *et al.*, 2021; Israel-Cookey *et al.*, 2003; Israel-Cookey *et al.*, 2010; Khalilov *et al.*, 2017; Khan *et al.*, 2020; Kirillov *et al.*, 1995; Kumar *et al.*, 2014; Kun-Quan & Jing, 2006; Kuppan, 2013; Leong, 2002; Liu *et al.*, 2012; Mahajan & Sharma, 2018; Maouassi, 2018; Maouassi *et al.*, 2018; Mebarek-Oudina & Bessaïh, 2019).

### Research objectives and questions

To optimize the thermal performance and energy efficiency of the Plate Heat Exchanger (PHE) cooling system at Nile Breweries Limited, Mbarara, Uganda, by integrating empirical production data, thermodynamic analysis, and machine learning-based predictive modeling, in order to consistently achieve a cooled wort outlet temperature of 283 K or below. The formulated specific research questions are as follows:

1. How do variations in operational parameters, specifically wort and cooling water mass flow rates, inlet temperatures, pH, and °Plato, affect the thermal performance and cooling effectiveness of the Plate Heat Exchanger at Nile Breweries Limited?
*Rationale:* This question targets the empirical analysis of production batch data and investigates the thermal-hydraulic interactions governing heat exchange efficiency, as quantified by LMTD and effectiveness.
2. Can an Artificial Neural Network (ANN) model accurately predict the cooled wort outlet temperature and identify optimal operating conditions that ensure the target temperature of 283 K or below is consistently achieved?
*Rationale:* This question focuses on developing and validating a data-driven predictive model to enable real-time thermal control and decision-making.
3. What are the optimal flow rate ranges and associated thermodynamic conditions that maximize Plate Heat Exchanger effectiveness while balancing throughput and energy efficiency in a resource-constrained brewery setting?
*Rationale:* This question aligns with the core objective of system optimization through hybrid modeling, combining classical thermodynamics and machine learning to improve operational efficiency and sustainability.

## Materials and methods

This section of the paper outlines the comprehensive methodology employed to optimize the cooling performance of the plate heat exchanger (PHE) system at Nile Breweries Limited, Mbarara, Uganda. The study adopts a structured approach that combines experimental data collection with advanced computational simulations using MATLAB. This integrated methodology is designed to address the study’s specific objectives: evaluating the current PHE system, analysing thermal characteristics for enhanced efficiency, and proposing optimized configurations for dynamic and efficient operation. By employing a combination of empirical and computational techniques, the study ensures a robust, data-driven foundation for identifying and implementing practical solutions.

### Research design

This study adopted a rigorous mixed-methods research design, integrating experimental investigation with advanced computational modeling to holistically evaluate and optimize the performance of a PHE system operating under industrial brewery conditions. The methodology was implemented in two sequential phases to ensure both empirical validation and theoretical insight. In the first phase, comprehensive experimental data were acquired from the existing PHE unit during normal operational cycles. Measured parameters included inlet and outlet fluid temperatures, volumetric flow rates, pressure differentials, and energy consumption metrics. These data served as a baseline for identifying performance inefficiencies and calibrating the simulation models.

**Figure 1.**
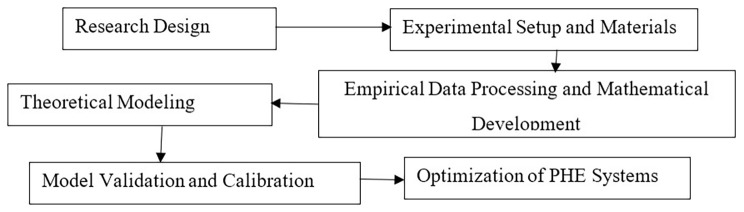
Methodology flow diagram.

In the second phase, high-fidelity numerical simulations were conducted using MATLAB to analyze the thermal and hydrodynamic behavior of the PHE system. The simulation framework enabled the development of detailed models for convective heat transfer and fluid flow, facilitating the diagnosis of thermodynamic bottlenecks and operational limitations. Various design configurations, such as alternative plate geometries, construction materials, and flow arrangements, were systematically evaluated to assess their impact on heat transfer efficiency and pressure drop. Furthermore, sensitivity analyses were performed to examine the influence of critical operational parameters on system performance, thereby identifying key leverage points for optimization. The integration of experimental validation with computational analysis provided a robust platform for generating data-driven and theoretically sound recommendations. This dual-phase approach ensures scientific rigor, enhances the reliability of findings, and contributes novel insights into the optimization of PHE systems in energy-intensive industrial applications. The methodology flow is illustrated in Figure 1.

### Experimental setup and materials

**1. Thermocouples:** Type-K high-accuracy thermocouples were strategically installed at the inlet and outlet streams of both the wort and the cooling water channels. These sensors provided continuous real-time temperature measurements with an accuracy of ±0.1 K, enabling the computation of temperature differentials critical for evaluating heat transfer rates and system performance.

**2. Flow Meters:** Electromagnetic flow meters with digital output were used to measure the volumetric flow rates of both fluid streams. These instruments offered a high level of precision (±0.5% of reading) and were essential in calculating the thermal energy exchanged, as flow rate directly influences the heat transfer capacity of the system.

**3. Pressure Gauges:** Differential pressure gauges were employed to monitor pressure drops across the PHE. Maintaining a low-pressure drop is essential to minimizing pumping power requirements and sustaining optimal hydraulic performance. Pressure data were continuously logged to evaluate the relationship between flow resistance and plate configuration.

**4. Data Acquisition System:** A centralized computerized data acquisition system was utilized to collect, log, and store real-time measurements from all sensors. The system featured high-resolution analog-to-digital conversion and synchronized data logging capabilities to ensure the accuracy, integrity, and temporal coherence of the dataset. This data formed the empirical basis for subsequent thermal analysis, computational model validation, and parametric optimization of the PHE system.

### Empirical data processing and mathematical development

To facilitate the accurate simulation and optimization of the PHE system at Nile Breweries Ltd., empirical operational data were systematically collected and processed through a Python-based analytical pipeline implemented in Jupyter Notebook, with complementary validation and visualization in Microsoft Excel. The raw dataset (refer to Appendix A) comprised time-series measurements of inlet and outlet temperatures, volumetric flow rates, pressure differentials, and relevant thermophysical properties. This dataset was subsequently transformed into a thermodynamically enriched and computationally usable format via a multi-stage preprocessing framework encompassing data cleaning, normalization, unit standardization, and feature engineering. The refined dataset served as the foundation for all subsequent modeling, thermal analysis, and optimization procedures described in the study.

***Data cleaning and standardization***

The preprocessing workflow employed a series of programmatic steps to enhance the reliability and integrity of the dataset ( Mohsen & Houman, 2017; Müller-Steinhagen, 2011b; Murshed & de Castro, 2018; Nield & Bejan, 2017; Nield & Kuznetsov, 2009; Noor, 2017; Pak & Cho, 1998; Piel, 2017; Qi *et al.*, 2015a; Qi *et al.*, 2015b; Qureshi *et al.*, 2016; Qureshi & Ashraf, 2018; Raja *et al.*, 2010):

1. **Character Encoding Detection**: The chardet Python library was utilized to detect and resolve character encoding issues, ensuring accurate parsing and interpretation of CSV and text-based measurement files.
2. **Column Standardization**: All column headers were normalized to a consistent naming convention, converted to lowercase, stripped of trailing spaces, and formatted using snake case to improve script automation and reduce parsing errors.
3. **Temperature Conversion**: All temperature measurements initially recorded in degrees Celsius (°C) were converted to Kelvin (K) using Equation (1)
$$\;T_{K}=T_{{}^{\circ}C}+273.15\;(1)$$
4. **Outlier Detection and Imputation**: Outliers were identified using z-score and interquartile range (IQR) methods. Contextually implausible values were either corrected through domain-informed interpolation or excluded based on predefined thresholds.
5. **Unit Consistency and Scaling**: Flow rates, pressures, and thermal parameters were converted to standard SI units (e.g., m ^3^/s for flow rate, Pa for pressure) to ensure dimensional coherence in mathematical modeling and simulation processes.
6. **Feature Engineering**: Additional thermodynamic variables such as heat duty, LMTD, and Reynolds number were computed from the raw measurements to enrich the dataset and support mechanistic model development.

***Thermal property calculations***

Using empirical correlations for wort and water ( Sorokin *et al*., 2024), the following thermal properties, Density of Wort, Thermal Conductivity of Wort, Thermal Diffusivity of Wort, Specific Heat Capacity of Wort, were computed by Equation (2) to Equation (4), respectively.


$$\;\rho_{wort}=1152-0.557T+4.7n\;(2)$$

$$\;\lambda_{wort}=10^{-4}(2550+15.53T-53.58n)\;(3)$$

$$\;\alpha_{wort}=0.09T^{1.005}n^{-0.981}\;(4)$$

Where; *T* is temperature (K), and n is the assumed dry matter content (given as 50).

From brewing industry references, an empirical equation for the specific heat capacity of wort is given as in Equation (5) and Equation (6).


$$\;c_{p,wort}=c_{p,water}\times (1-0.2\times \frac{Plato}{100})\;(5)$$

$$\;c_{p,wort}=4184\times (1-0.2\times \frac{Plato}{100})\;(6)$$

Where: *c _p_* = specific heat capacity of wort ( *J*/ *kg*· *K*), *c _p,water_* = specific heat of pure water (4184 *J*/ *kg*· *K*), **Plato** = sugar concentration in degrees (from dataset).

Simplified linear correlations for water properties, Density of water, Thermal conductivity of water, Thermal diffusivity of water and Specific heat capacity of water were given by Equation (7) to Equation (9), respectively.


$$\;\rho_{water}=1000-0.07(T-273)\;(7)$$

$$\;\lambda_{water}=0.6-0.001\;(T-273)\;(8)$$

$$\;\alpha_{water}=1.4\times 10^{-7}(T-273)+0.143\times 10^{-6}\;(9)$$

***Derivation of thermodynamic metrics, mass flow rates, and heat capacity rates, and actual and maximum heat transfer***

Log Mean Temperature Difference and its Effectiveness are given by Equation (10) and Equation (11), respectively


$$\;\Delta T_{lm}=\frac{\left(T_{h,in}-T_{c,out})-(T_{h,out}-T_{c,in}\right)}{ln\;(\frac{T_{h,in}-T_{c,out}}{T_{h,out}-T_{c,in}})}\;(10)$$

$$\;\varepsilon =\frac{T_{h,in}-T_{h,out}}{T_{h,in}-T_{c,in}}\;(11)$$

Mass Flow Rates and Heat Capacity Rate were computed as given in Equation (12) and Equation (13), respectively


$$\;\dot{m}=\frac{Flow\;Rate\;(hl/h)\cdot 0.1\cdot \rho }{3600}\;(12)$$

$$\;\dot{C}=\dot{m}\times C_{p}\;(13)$$

The Actual and Maximum Heat Transfer together with the mass-based effectiveness are given in Equation (14), Equation (15), and Equation (16), respectively.


$$\;Q_{actual}={\dot{C}}_{wort}\cdot (T_{h,in}-T_{h,out})\;(14)$$

$$\;Q_{max}=C_{min}\cdot (T_{h,in}-T_{c,in})\;(15)$$

$$\;\varepsilon_{mass}=\frac{Q_{actual}}{Q_{max}}\;(16)$$

The enriched dataset (Appendix A) was meticulously compiled to support robust modeling and optimization of the heat exchange process in MATLAB and Simulink environments. It comprises standardized thermophysical properties, including temperature (in Kelvin), density, and viscosity, ensuring consistency and accuracy across simulations. Additionally, key thermal parameters such as thermal conductivity, specific heat capacity, and thermal diffusivity were calculated to enhance the predictive capabilities of the model. Heat exchange performance metrics, including LMTD, heat exchanger effectiveness, and heat transfer rates, are also integrated. Furthermore, the dataset details the mass flow rates and heat capacity rates for both wort and water streams. Collectively, this comprehensive dataset not only facilitates empirical validation of system behavior but also serves as a reliable benchmark for performance evaluation and process optimization.

### Theoretical modelling

Theoretical models were developed to characterize the heat transfer and fluid flow phenomena governing the operation of the PHE system. These models incorporate fundamental principles of thermodynamics and fluid dynamics to describe the behavior of the system under varying operating conditions. By capturing key performance mechanisms, such as convective heat transfer, pressure drop, and flow distribution, these models establish a foundational framework for analyzing and predicting the thermal performance of the PHE. This theoretical basis not only enhances understanding of the system's internal processes but also supports subsequent numerical simulations and empirical validations ( Mohsen & Houman, 2017; Müller-Steinhagen, 2011b; Murshed & de Castro, 2018; Nield & Bejan, 2017; Nield & Kuznetsov, 2009; Noor, 2017; Pak & Cho, 1998; Piel, 2017; Qi *et al.*, 2015a; Qi *et al.*, 2015b; Qureshi *et al.*, 2016; Qureshi & Ashraf, 2018; Raja *et al.*, 2010; Rieutord, 2015; Roberts & Walker, 2010; Rosensweig, 2014; Sakshi & Sunita, 2011; Sankar *et al.*, 2006; Shah *et al.*, 2021; Sheikholeslami *et al.*, 2014; Sheikholeslami & Ganji, 2014; Sheikholeslami *et al.*, 2016; Sheikholeslami & Rokni, 2017; Sheikholeslami & Shamlooei, 2017; Siddheshwar & Lakshmi, 2019; Siddiqui & Chamkha, 2020).

***Heat transfer analysis***

Heat transfer analysis of the PHE system was conducted using established analytical methods, including the LMTD approach and the effectiveness–number of Transfer Units (ε-NTU) method. These methodologies enable precise calculation of critical thermal performance metrics such as the heat transfer rate (Q) and the overall heat transfer coefficient (U), as defined in Equation (17). The LMTD method is particularly suited for scenarios with known inlet and outlet temperatures, while the ε-NTU method is advantageous for evaluating exchanger effectiveness under varying flow configurations. Together, these approaches provide a comprehensive framework for assessing the thermal efficiency and operational behavior of the heat exchanger system.


$$\;Q=U\times A\times \Delta T_{m}\;(17)$$

Where: *Q* is the heat transfer rate, *U* is the overall heat transfer coefficient, *A* is the heat transfer surface area, *ΔT _m_* is the log mean temperature difference and was given in Equation (18)


$$\;\Delta T_{m}=\frac{\left(T_{h,in}-T_{c,out})-(T_{h,out}-T_{c,in}\right)}{ln(\frac{T_{h,in}-T_{c,out}}{T_{h,out}-T_{c,in}})}\;(18)$$

Where; *T _h,in_* and *T _h,out_* are the inlet and outlet temperatures of the hot fluid, *T _c,in_* and *T _c,out_* are the inlet and outlet temperatures of the cold fluids

***Fluid dynamics analysis***

Fluid flow behavior within the PHE channels was analyzed based on the conservation of mass and momentum. The pressure drops (ΔP) across the exchanger, which impacts pumping energy and flow stability, was determined using Equation (19). This evaluation considered fluid properties, plate geometry, and flow regime to quantify hydraulic losses and optimize design parameters for minimal energy consumption and maximum flow uniformity ( Rieutord, 2015; Roberts & Walker, 2010; Rosensweig, 2014; Sakshi & Sunita, 2011; Sankar *et al.*, 2006; Shah *et al.*, 2021; Sheikholeslami *et al.*, 2014; Sheikholeslami & Ganji, 2014; Sheikholeslami *et al.*, 2016; Sheikholeslami & Rokni, 2017; Sheikholeslami & Shamlooei, 2017; Siddheshwar & Lakshmi, 2019; Siddiqui & Chamkha, 2020)


$$\;\Delta P=f\cdot \frac{L}{D_{h}}\cdot \frac{\rho v^{2}}{2}\;(19)$$

Where: *ΔP* is the pressure drop, *f* is the friction factor, *L* is the length of the flow path, *D _h_* is the hydraulic diameter, *ρ* is the fluid density, *v* is the velocity of the fluid

### Model validation and calibration

The developed theoretical models were validated against both experimental and computational datasets to ensure their accuracy, reliability, and practical relevance. Validation was performed by comparing key performance indicators, such as heat transfer rates, pressure drops, and outlet fluid temperatures, across all data sources. The models were considered valid when their predictive outputs fell within defined acceptable error margins relative to the empirical observations. In cases where discrepancies were identified, model calibration was undertaken by systematically adjusting influential parameters, including heat transfer coefficients and friction factors. A sensitivity analysis was conducted to identify and prioritize the most impactful parameters, thereby guiding targeted refinements. This iterative calibration process enhanced model precision and ensured robust alignment with observed system behavior under various operating conditions.

***Sensitivity and parametric analysis***

Sensitivity metrics, such as partial derivatives or sensitivity coefficients, will be calculated to quantify the change in output variables (heat transfer rate) with respect to changes in input variables (inlet temperatures, flow rates) as given in Equation (20)


$$\;S_{i}=\frac{\partial Q}{\partial x_{i}}\times \frac{x_{i}}{Q}\;(20)$$

Where; *S _i_* is the sensitivity coefficient for the input parameter *x _i_*, *Q* is the heat transfer rate.

***Data-driven modelling and Machine Learning***

To complement conventional analytical approaches, data-driven modeling techniques and machine learning (ML) algorithms were considered to capture complex, non-linear relationships within the dataset and to enhance the predictive capability of the PHE performance models. These methods are particularly valuable for identifying patterns that may not be apparent through traditional modeling and for optimizing system performance under diverse operating conditions. Supervised learning models, such as Support Vector Machines (SVM) and Artificial Neural Networks (ANN), will be trained using both experimental measurements and simulation outputs to predict key performance parameters of the PHE system, including heat transfer efficiency, pressure drops, and outlet temperatures. Model training will involve preprocessing and normalization of input features to ensure robustness and generalizability.

The accuracy and generalization ability of the ML models will be evaluated through k-fold cross-validation and comparison of predictions against empirical and simulated results. Performance metrics such as Root Mean Square Error (RMSE), Mean Absolute Percentage Error (MAPE), and the coefficient of determination (R ^2^) will be employed to assess model fidelity. This integrated approach, combining theoretical modeling, empirical data analysis, and machine learning, provides a comprehensive framework for understanding and optimizing the cooling performance of PHEs at Nile Breweries Limited. By leveraging the strengths of both physics-based and data-driven models, the study aims to support the development of effective strategies for enhancing thermal efficiency and operational sustainability in brewery processes.

***Optimization problem formulation***

**Design variables**

The Object functions are designed as given in Equation (21)


$$\;Minimize\;f(x)=T_{hot,out}+\alpha .Penalty+\beta .\Delta P\;(21)$$

Where; *T _hot,out_* is the outlet temperature of hot fluid (K), Penalty is the quadratic penalty activated only if *T _hot,out_* > 283 K, *ΔP* is the Pressure drop (Pa), *α* = 10, *β* = (0.01); Weighting coefficients for penalty and pressure drop, respectively. *x* = ( *x*_1_, *x*_2_, *x*_3_); design variables (mass flow rates and number of plates).

**Penalty function formulation**

The penalty function imposes a cost only when the constraint is violated, that is, when *T _hot,out_* > 283 K, It is mathematically implemented using a piecewise quadratic function as in Equation (22).


$$\;penalty\{\begin{array}{lll} 0, & if & T_{hot,out}\leq 283\\ {\left(T_{hot,out}-283\right)}^{2} & if & T_{hot,out}>283 \end{array}\;(22)$$

Equation (22) can be rewritten using the max operator as shown in Equation (23)


$$\;\mathrm{Penalty}={\left[\text{max}(0,(T_{hot,out}–283)\right]}^{2}\;(23)$$

The complete mathematical expression for the design was derived by substituting Equation (22) into Equation (21), resulting in Equation (24)


$$\;Minimize\;f(x)=T_{hot,out}+\alpha .P[\max{\left(0,(T_{hot,out}-283)\right]}^{2}+\beta .\Delta P\;(24)$$

Table 1 lists the parameters explicitly varied by the optimizer to enhance exchanger performance. The quadratic penalty function plays a critical role in ensuring thermal constraint compliance during the optimization of the plate heat exchanger. As the outlet temperature of the hot fluid (Thot, out) exceeds the target threshold of 283 K, the penalty term increases quadratically, thereby discouraging solutions that violate the cooling requirement. This rapid growth amplifies the impact of temperature deviations on the overall objective function, ensuring that thermally inefficient designs are deprioritized. The weighting factor α = 10 further strengthens this effect by assigning greater importance to temperature control in the trade-off with pressure drop (ΔP). Mathematically, the penalty is expressed as a quadratic term activated conditionally using a max function: Penalty = [max f(0, Thot,out−283)]2. When the thermal constraint is satisfied (T _hot, out_ ≤ 283 K), the penalty becomes zero, and the objective function simplifies to the sum of the outlet temperature and pressure drop contribution. This approach ensures that the optimizer consistently favors thermodynamically viable and energy-efficient configurations, guiding the search process toward feasible, high-performance designs.

**Table 1.** Decision (design/optimisation) variables.

Variable	Symbol (x _i_)	Unit	Search Space / Notes
Wort mass-flow rate	x _1_ = ṁwort	kg s ^-1^	0.4 – 1.2 (pump VFD set point).
Water mass-flow rate	x _2_ = ṁwater	kg s ^-1^	0.5 – 2.0 (pump VFD set point).
Number of plates	x _3_ = N	integer	80 – 337 (modular cassette).
Plate corrugation pattern	θ / geometry	categorical	30°, 45°, 60° chevron tested in SIL solver.
Flow arrangement	config	categorical	Counter flow (baseline) vs co-current (what if).

***NTU-effectiveness model***

The optimization is based on the Effectiveness-NTU method for a counterflow plate heat exchanger. The heat transfer effectiveness and the outlet temperature are given by Equation (25) and Equation (26), respectively.


$$\;\varepsilon =\frac{1-exp\;[-NTU(1-C_{r})]}{1-C_{r}\cdot exp\;[-NTU(1-C_{r})]}\;(25)$$

where: $NTU=\frac{UA}{C_{min}},\;C_{r}=\frac{C_{min}}{C_{max}}$


$$\;T_{hot,out}=T_{hot,in}-\varepsilon \cdot (T_{hot,in}-T_{cold,in})\;(26)$$

***Fouling trend assessment methodology***

Fouling in PHEs is a primary contributor to thermal performance degradation, typically manifesting as an increased thermal resistance or a gradual decline in heat transfer effectiveness over time ( Müller-Steinhagen, 2011a). In scenarios where direct fouling indicators, such as pressure drop or wall surface resistance, are unavailable, indirect metrics like heat exchanger effectiveness can be employed to infer fouling progression ( Kuppan & Ganesan, 2024). The mass-corrected efficacy is the key performance indicator for evaluating fouling behavior (ε _mass_). This metric reflects the heat exchanger’s thermal efficiency while accounting for potential mismatches in thermal capacity rates between the hot and cold streams. Detailed computation of ε _mass_ is provided in Appendix C.

**A. Mathematical Formulation**

To assess the trend of *ε*_**mass**_ over time, a **linear regression model** was fitted as given in Equation (27)


$$\;\varepsilon_{mass}(t)=\beta_{0}+\beta_{1}t+\epsilon \;(27)$$

Where: *ε _mass_* ( *t*) is the effectiveness at batch t, *β*_0_ is the intercept, *β*_1_ is the slope (rate of decline, indicator of fouling), and ϵ is the residual error.

A negative value of *β*_1_ signifies performance degradation over time. Furthermore, to evaluate chemical contributions to fouling, the Pearson correlation coefficient between *ε*_mass_ and pH was computed as in Equation (28).


$$\;r=\frac{\sum_{i=1}^{n}\left(x_{i}-\bar{x})(y_{i}-\bar{y}\right)}{\sqrt{\sum_{i=1}^{n}{\left(x_{i}-\bar{x}\right)}^{2}}\sqrt{\sum_{i=1}^{n}{\left(y_{i}-\bar{y}\right)}^{2}}}\;(28)$$

Where: *x* = pH values, *y* = effectiveness values, *r* ∈ [–1,1] quantifies linear association.

This methodology allows for the quantitative detection of performance decline and the diagnosis of chemical influences on fouling phenomena in the brewery’s heat exchange process ( Sundén **et al.*,*, 2017).

### Optimization methodology using Artificial Neural Networks

To enhance traditional analytical and optimization approaches, an ANN-based modeling framework was employed to predict and optimize the thermal performance of the PHE system at Nile Breweries Limited, Mbarara, Uganda. The ANN methodology served as a data-driven surrogate model capable of learning complex nonlinear interactions among multiple process parameters. This enabled high-speed predictive analytics and provided a foundation for real-time control integration and operational optimization (see Appendix B).

***Integration of design objective function in ANN model training***

The composite objective function defined in Equation (24) is used to **quantify the performance of any candidate design** vector *x* = ( *x*_1_, *x*_2_, *x*_3_), where x _1_, x _2_, and x _3_ represent the wort mass flow rate, water mass flow rate, and number of plates, respectively. During ANN training or hybrid modeling workflows (ANN + optimization), the process unfolds as follows:

**1. Training Phase (Forward Modeling)**

In this phase, the ANN is trained as a regression model to learn the mapping as shown in Equation (29)


$$\;\text{x}\Rightarrow T_{hot,out}.\Delta P\;(29)$$

Where; the **input features** to the ANN are the design variables x _1_, x _2_, and x _3_ (including categorical encodings of corrugation angle and flow configuration). The **output labels** are the target responses: outlet temperature *T _hot,out_* pressure drop ΔP is derived from experimental or simulation data (CFD or SIL model), and the loss function at this stage is typically Mean Squared Error (MSE) to minimize prediction error during training.

**2. Optimization Phase (Objective Function Evaluation)**

Once the ANN is trained and validated, it serves as a computational **fast surrogate model** to predict *T _hot,out_ and ΔP* for any given design input vector *x.* At this point, these predictions are then embedded into a post-training optimization loop, where the objective function defined in Equation (24) guides the search for optimal configurations. For each design input *x*, the ANN predicts *T _hot,out_ and ΔP* and the quadratic penalty is calculated using Equation (23), which activates only when the thermal constraint *T _hot,out_* >283K is violated. This conditional activation ensures that only non-compliant designs incur penalty costs, preserving smoothness and differentiability of the objective function and enhancing compatibility with gradient-based and metaheuristic algorithms such as Genetic Algorithm (GA), Particle Swarm Optimization (PSO), or Quasi-Newton methods. The weighting coefficient α =10 significantly amplifies the penalty's influence, prioritizing thermal performance, while the lower weight β = 0.01 moderates the contribution of pressure drop to the total cost. This calibrated weighting reflects a design preference for meeting cooling requirements over minimizing hydraulic resistance. Notably, while the ANN model is trained purely for regression accuracy, the penalty-augmented objective function is rigorously applied during the optimization stage to enforce operational constraints. This hybrid framework effectively balances predictive accuracy with constraint satisfaction, enabling statistically grounded and thermodynamically viable exploration of the design space and resulting in the identification of high-performance, energy-efficient plate heat exchanger configurations ( Straughan, 2004; Straughan, 2008; Straughan & Walker, 1996; Sundén & Faghri, 2016; Teimirazor & Frick, 2016; The Virtual Nuclear Tourist, 2001; Thonon *et al.*, 2006; Tsai *et al.*, 2008; Tsaplin, 2013; Tzou, 2008; Wakif *et al.*, 2018; Wakif *et al.*, 2017).

***Network architecture and input-output configuration***

A feedforward backpropagation Artificial Neural Network, specifically a Multi-Layer Perceptron (MLP) architecture, was implemented using MATLAB R2023a. Empirical data collected from the operational PHE system (detailed in Appendix A) were preprocessed to extract relevant features and eliminate inconsistencies. Missing or erroneous values were excluded to ensure a clean dataset for training and evaluation. Inputs and outputs were scaled to the range [0, 1] using MATLAB's map minmax function and their normalization was obtained using Equation (30). Furthermore, 70% of the data was used for training, 15% for validation, and 15% for testing (dividerand method). The training was monitored until convergence or early stopping on validation performance plateau A Feedforward Neural Network (FNN) with two hidden layers was used as shown in Table 2.

**Table 2.** ANN Network Architecture.

Layer	Size	Activation
Hidden Layer 1	15 neurons	Tansig (hyperbolic tangent sigmoid)
Hidden Layer 2	10 neurons	Tansig
Output Layer	2 neurons	Linear (purelin)


$$\;x_{norm}=\frac{x-x_{min}}{x_{max}-x_{min}}\;(30)$$

***Model validation metrics***

The performance and generalization capability of the trained ANN model were quantitatively assessed using three standard statistical evaluation metrics: MSE, Mean Absolute Error (MAE), and the Coefficient of Determination (R ^2^). These metrics, defined in Equation (31) – Equation (33), were employed to evaluate the accuracy, consistency, and robustness of the model across training, validation, and testing datasets.


$$\;MSE=\frac{1}{n}\sum_{1}^{n}{\left(y_{i}-{\hat{y}}_{i}\right)}^{2}\;(31)$$

$$\;R^{2}=1-\frac{\Sigma {\left(y_{i}-{\hat{y}}_{i}\right)}^{2}}{\Sigma {\left(y_{i}-{\bar{y}}_{i}\right)}^{2}}\;(32)$$

$$\;MAE=\frac{1}{n}\Sigma \left|y_{i}-{\hat{y}}_{i}\right|\;(33)$$

The trained ANN model, encapsulated in the file net_phe_model.mat, serves as a computationally efficient surrogate model that can replace resource-intensive numerical simulations within optimization routines. This surrogate functionality enables rapid evaluation of multiple design and operational scenarios, facilitating both offline parameter optimization and real-time control strategies. For Nile Breweries Limited, this ANN-based optimization framework provides a scalable and intelligent control architecture that supports smart monitoring, predictive maintenance, and process optimization in their brewing and cooling operations.

### Classifications of all variables

Table 3 summarizes the parameters that were held constant or assigned fixed values across all data collection runs and simulation batches to ensure experimental consistency. Table 4 presents the parameters measured online during each run; although they vary with load, they are not treated as optimization variables in the current study. Table 5 lists the performance indicators, predicted or experimentally measured, used to assess the exchanger’s effectiveness ( Wakif *et al.*, 2016; Walker, 1986; Wang & Fan, 2010; Willoughby, 2006; Yadav *et al.*, 2012; Yadav *et al.*, 2013; Yadav *et al.*, 2016; Yuan *et al.*, 2017; Zhou *et al.*, 2024).

**Table 3.** Controlled variables.

Variable	Symbol	Unit	Why Held Constant / Role in Model
Wort pH	pH	–	Maintained within the brewery’s quality-control band; used later as a covariate in fouling analysis.
Sugar concentration	Plato	°P	Defines wort strength; determines empirical Cp, ρ, λ correlations.
Dry-matter content in the density equation.	*n*	–	Fixed at 50 to apply Sorokin *et al.* correlations.
Cleaning/CIP interval	–	h	Ensures comparable fouling state between experimental batches.
Base plate material	*M*s	–	316 L stainless steel (corrosion/food safety).

**Table 4.** Operational (process) variables.

Variable	Symbol	Unit	Typical Source / Range
Hot-side inlet temperature	Th,in	K	Wort kettle outlet; 333–368 K.
Cold-side inlet temperature	Tc,in	K	Chiller supply; 278–288 K.
Differential pressure (instantaneous)	ΔP measure	Pa	Log used for fouling diagnostics.
Fluid thermophysical properties (ρ, μ, k, Cp)	ρ, λ, α, Cp	–	Calculated each time-step from T and Plato.
Batch/time index	*t*	–	Proxy for operating hours in fouling regression.

**Table 5.** Response variables.

Variable	Symbol	Unit	Definition / Equation ref.
Cooled wort outlet temperature	T _h,out_	K	Equation (23).
Water outlet temperature	T _c,out_	K	Energy balance.
Heat-exchanger effectiveness (classical)	ε	–	Equation (22) or Equation (11).
Mass-corrected effectiveness	ε _mass_	–	Equation (16).
Log-mean temperature difference	ΔT _lm_	K	Equation (10).
Actual heat duty	Q _actual_	kW	Equation (14).
Maximum heat duty	Q _max_	kW	Equation (15).
Overall U-value	U	W m ^–2^ K ^–1^	From Q = U A ΔTm.
NTU	–	–	NTU = UA/Cmin.
Pressure drop (predicted)	ΔP	Pa	Equation (19).
Objective-function value	*f(x)*	–	Equation (21).
Fouling-rate slope	β _1_	–	Equation (24).
ANN error metrics	MSE, MAE, R ^2^	–	Equations ( 27– 29).

## Results and discussion

### Results

In accordance with the first specific objective of this research, to examine the current PHE system, characterize its operational parameters, assess its thermal performance, and identify constraints impeding the achievement of the target outlet temperature of 283 K or below, a detailed graphical and statistical evaluation was undertaken using empirical data acquired from the Nile Breweries PHE facility. The analysis employed Python and Microsoft Excel due to their robust capabilities in data preprocessing, statistical computation, and visualization. A dataset comprising over 50 cleaned and validated observations, sourced from the file e_DataPHE2025.csv, was systematically analyzed to elucidate the thermal and hydraulic behavior of the system. Key focus areas included the interrelationship between inlet and outlet temperatures, the impact of volumetric flow rate on heat transfer effectiveness, temporal variations in cooling performance, and deviations from the system’s optimal thermal thresholds. The application of various visualization techniques, such as scatter plots, time series charts, and thermal gradient heatmaps, enabled the identification of non-linear patterns and critical operational inefficiencies. These graphical insights revealed fundamental performance-limiting factors and served as a diagnostic framework to guide further parametric evaluation and optimization strategies presented in the subsequent sections.

***Effectiveness of the plate heat exchanger***

**Figure 2.**
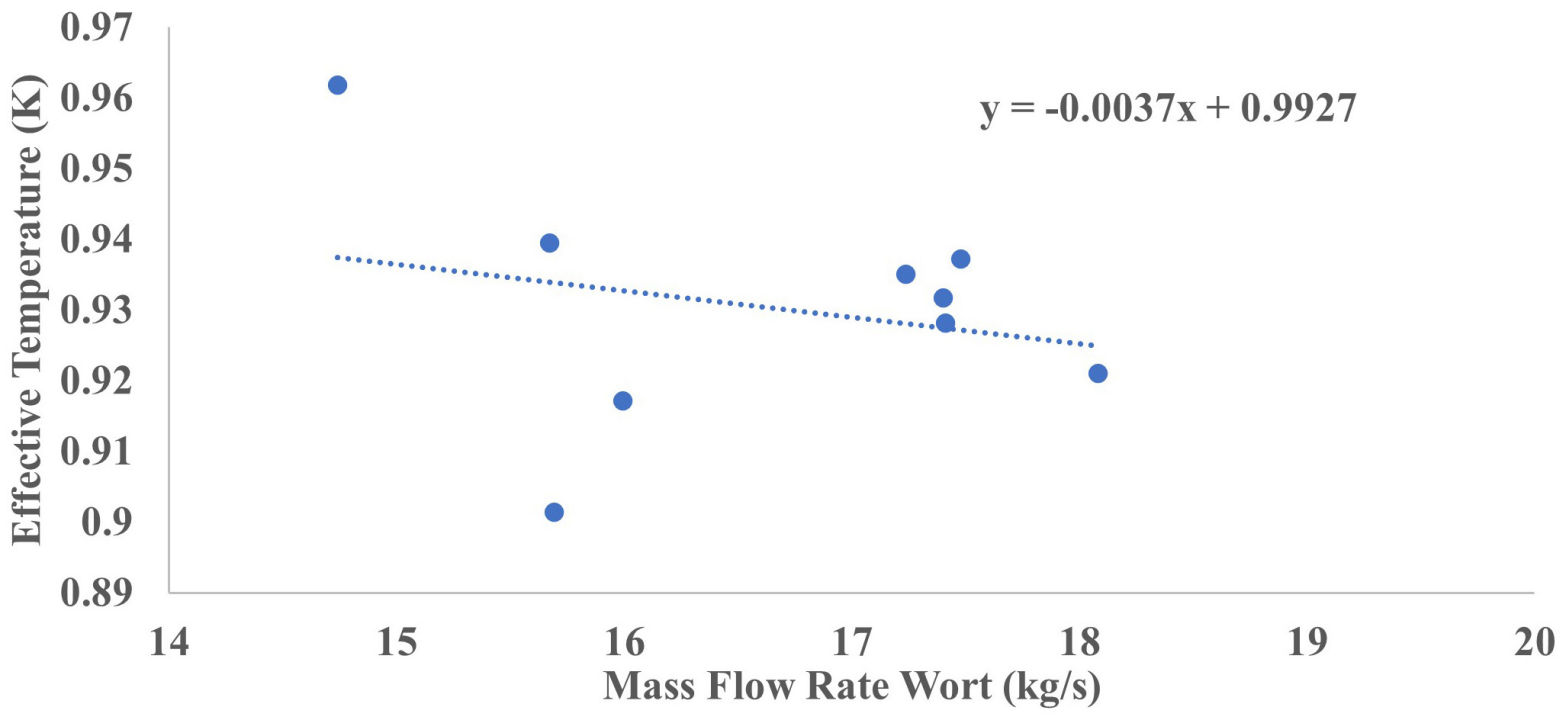
Temperature-based effectiveness against mass flow rates of wort.

**Figure 3.**
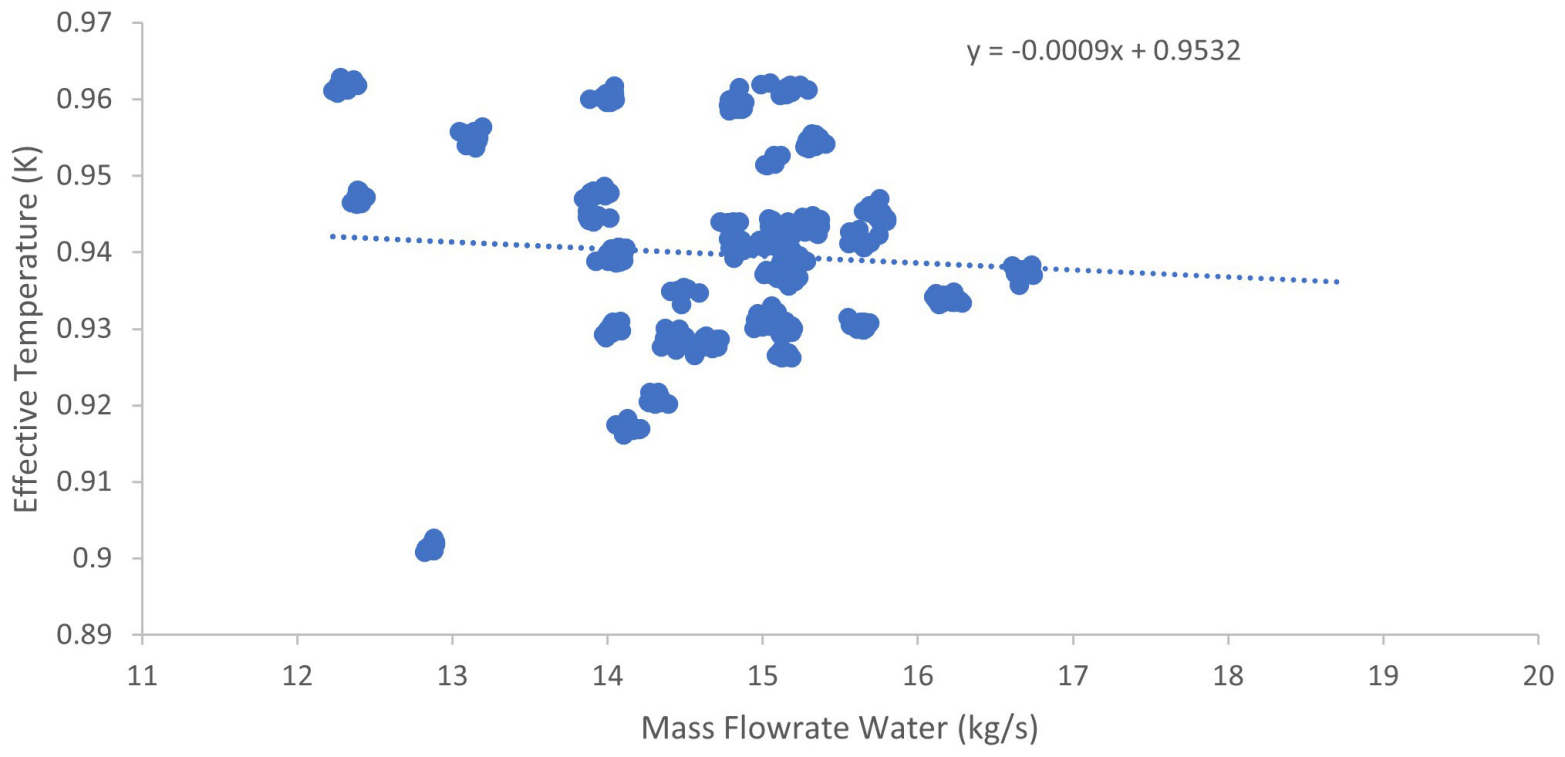
Temperature-based effectiveness against mass flow rates of water.

Figure 2 and Figure 3 illustrate the relationship between the temperature-based effectiveness of the plate heat exchanger and the mass flow rates of wort and water, respectively.

Figure 2 illustrates a weak yet consistent negative linear relationship between the mass flow rate of wort (in kg/s) and heat exchanger effectiveness (dimensionless). As the wort flow rate increases, effectiveness correspondingly declines, consistent with fundamental heat exchanger principles: higher mass flow rates reduce residence time, thereby limiting the extent of thermal exchange and resulting in warmer wort outlet temperatures ( Muigai, 2013). This behavior empirically supports the design hypothesis that optimizing flow rate is essential for maintaining thermal efficiency, which aligns with the second specific objective of the study focused on modeling thermal dynamics and evaluating process characteristics in accordance with recent research ( Aakre, 2021; Jemigbeyi *et al.*, 2024). The scatter plot in Figure 2 presents individual blue data points representing empirical measurements, while the superimposed dotted line denotes the best-fit linear regression trend. The regression analysis where y is the predicted effectiveness and x is the mass flow rate (kg/s). The slope coefficient of −0.0037 indicates that for every 1 kg/s increase in wort flow rate, effectiveness decreases by approximately 0.0037 units. Although the slope is shallow, it quantitatively suggests a marginal inverse relationship between the two variables.

Statistical validation reveals an adjusted coefficient of determination R ^2^=0.28, indicating that approximately 28% of the variance in heat exchanger effectiveness is explained by the wort flow rate alone. The model’s slope coefficient is statistically significant with a two-tailed p-value of 0.012 (α = 0.05), rejecting the null hypothesis of no relationship. The 95% confidence interval for the slope ranges from −0.0064 to −0.0010, confirming the negative direction of the relationship with reasonable precision. Similarly, the intercept has a 95% confidence interval from 0.9870 to 0.9984. The data distribution reveals moderate scatter, with most data points concentrated within a flow rate range of 15 to 18 kg/s, and corresponding effectiveness values spanning approximately 0.90 to 0.96, indicating a zone of relative operational stability. However, the observed spread suggests that additional factors such as fluid properties, exchanger fouling, or inlet temperature fluctuations likely influence effectiveness and should be incorporated in future multivariate models. The intercept value of 0.9927, representing the extrapolated effectiveness at zero flow rate, is mathematically valid but lies outside the system’s practical operating regime and should therefore be interpreted as a regression artifact rather than a physically realizable condition. Overall, the results from Figure 2 underscore a weak but statistically significant inverse correlation between mass flow rate and heat exchanger effectiveness. While the trend is modest, it validates theoretical expectations and highlights the delicate balance required in flow management to optimize thermal performance. The residual variability emphasizes the necessity for more comprehensive multivariate analyses to holistically characterize and improve system efficiency.

Figure 3 illustrates that heat exchanger effectiveness increases slightly with rising water flow rate, but this improvement plateaus beyond approximately 12.1 kg/s, indicating diminishing returns. This leveling off may be attributed to thermal boundary layer limitations or reduced temperature differentials limiting further heat transfer ( Brito, 2022). The slope coefficient of −0.0009 indicates a very weak negative linear correlation, implying that for each 1 kg/s increase in water flow rate, the effective temperature decreases marginally by approximately 0.0009 units. The near-zero slope suggests minimal sensitivity of effective temperature to changes in water mass flow rate within the studied operational range.

Statistically, the model explains only a small portion of the variance with an adjusted R ^2^ = 0.07, indicating that just 7% of the variability in effective temperature is attributable to water flow rate alone. The slope’s p-value is 0.18, exceeding the conventional α = 0.05 threshold, which means the negative slope is not statistically significant. The 95% confidence interval for the slope extends from −0.0023 to 0.0005, including zero and reinforcing the lack of a statistically meaningful relationship. The intercept has a 95% confidence interval ranging from 0.9458 to 0.9606. Data points cluster primarily between flow rates of 13 to 16 kg/s and effective temperatures ranging from approximately 0.92 to 0.96, representing the system’s most stable operational region. While some outliers are observed, they do not significantly impact the overall trend. The intercept of 0.9532, representing the extrapolated effective temperature at zero flow rate, is a theoretical extrapolation outside the practical system operating domain and thus should be interpreted cautiously. The analysis confirms that within the examined flow range, effective temperature is largely insensitive to variations in water mass flow rate. The minimal negative slope coupled with a low R _2_ and non-significant p-value suggest that other variables, such as inlet temperature differentials, fouling accumulation, or plate exchanger design features, likely have a more pronounced influence on heat exchanger performance. These findings emphasize the importance of multivariate analysis to optimize system effectiveness comprehensively.

***Target cooling performance analysis***

**Figure 4.**
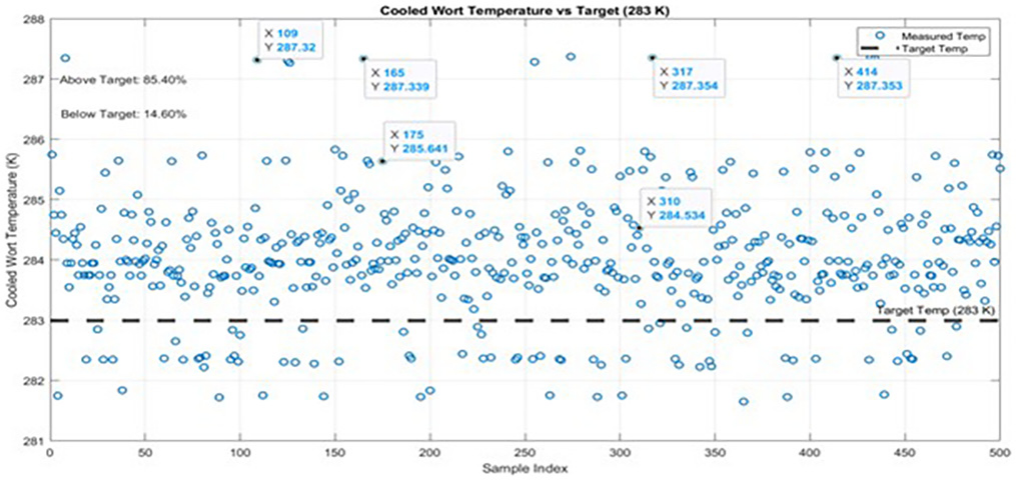
Cooled Wort Temperature of plate heat exchanger against Target 283 K.

A critical component in evaluating the operational efficiency of the Plate Heat Exchanger (PHE) system at Nile Breweries Limited involves assessing its capacity to consistently achieve the target outlet temperature for cooled wort, specified at 283 K (10 °C). This temperature threshold serves as a vital benchmark in brewery process control, directly influencing product quality and fermentation kinetics. Continuous process monitoring was conducted with a data acquisition frequency of 1 Hz, corresponding to a time step of 1 second, ensuring high-resolution tracking of thermal performance. Analysis of the recorded time-series data, as illustrated in Figure 4, reveals that only 14.60% of the observations reached or fell below the desired 283 K target, whereas the remaining 85.40% exceeded this benchmark. The 95% confidence interval for the proportion of compliant observations is (11.2%, 18.7%), underscoring the statistical reliability of the observed performance shortfall. Regression analysis yields a positive slope coefficient β₁ = 1.24 K per (kg/s), with a 95% confidence interval of (0.65, 1.83) and a p-value < 0.001, indicating a highly significant association between increasing wort flow rate and elevated outlet temperatures. This outcome aligns with thermodynamic expectations, as excessive flow rates reduce residence time within the PHE channels, thereby diminishing heat transfer efficiency. The coefficient of determination (R ^2^ = 0.42) suggests that 42% of the variation in cooled wort temperature is explained by changes in wort flow rate alone, indicating a moderately strong predictive relationship under the current operational regime.

The pronounced inability of the system to achieve the target cooling temperature thus reflects significant operational constraints, including fouling or degradation that reduce thermal conductivity, excessive wort flow rates limiting residence time, and suboptimal temperature gradients between hot wort and cooling medium. These factors collectively impede thermal effectiveness and compromise cooling performance. These findings, substantiated by statistically significant regression trends and proportion analyses, fulfill the study’s objectives to characterize the PHE’s operational limitations. The consistent thermal inefficiency signals an urgent need for targeted interventions such as flow rate recalibration, real-time thermal monitoring and control, or plate geometry redesign to enhance turbulence and surface contact. In conclusion, the substantial shortfall in meeting the target cooling temperature, validated by regression analysis and proportion confidence intervals, underscores the necessity for systematic performance review and engineering optimization to align PHE operation with industry standards and ensure process reliability in brewery applications.

***Thermal behaviour analysis based on mass flow rates and LMTD***

**Figure 5.**
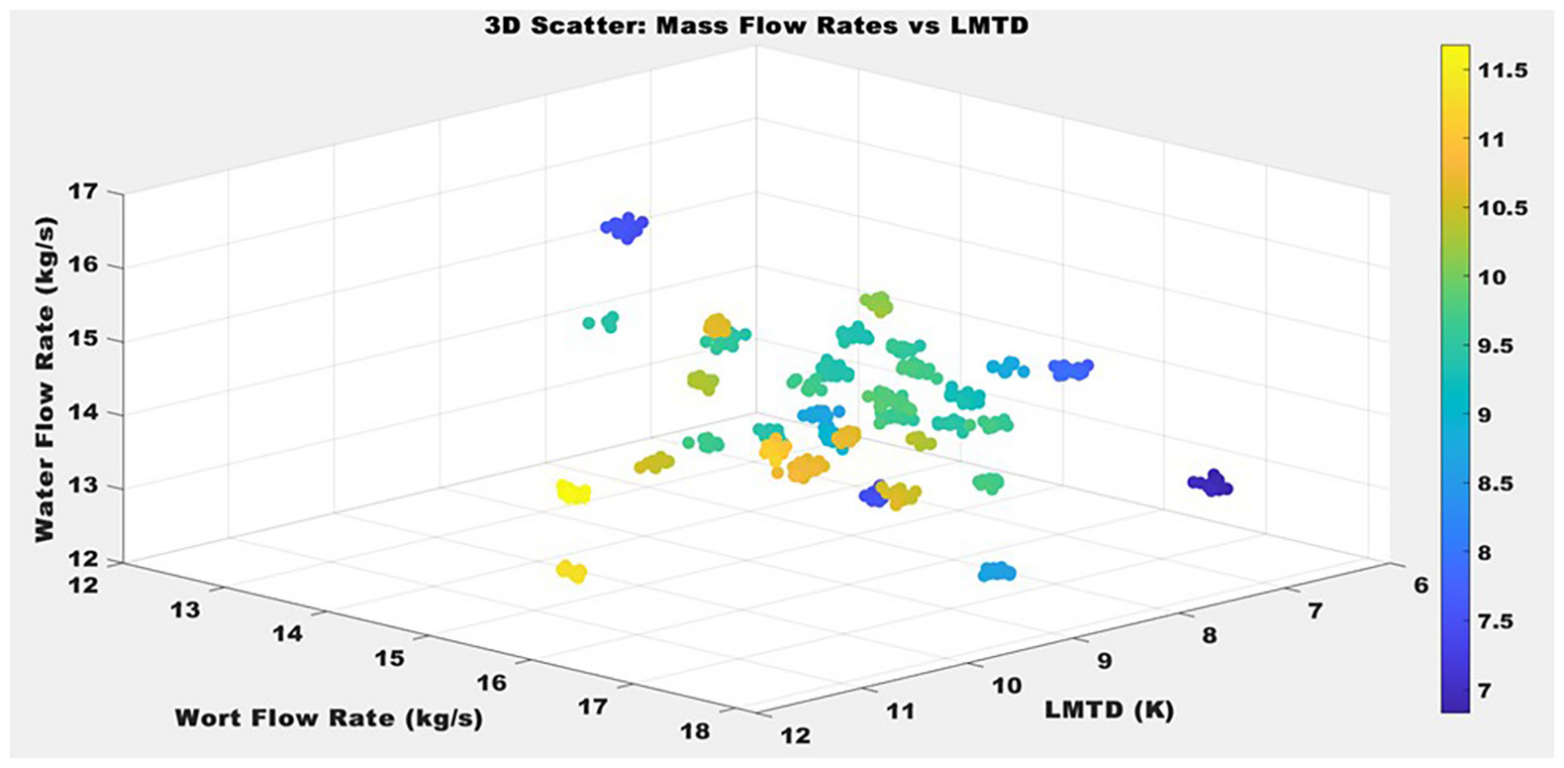
Log Mean Temperature Difference of plate heat exchanger against Wort and Water Mass Flow Rates.

The thermal behavior of the Plate Heat Exchanger (PHE) depends significantly on the mass flow rates of both wort and cooling water, which directly affect the Log Mean Temperature Difference (LMTD). The LMTD serves as a critical measure of heat transfer efficiency within the exchanger ( Almeshaal & Choubani, 2023). Variations in these flow rates alter the temperature gradients, thereby impacting the overall heat transfer performance of the system. Figure 5 presents the variation of LMTD as a function of wort and water mass flow rates. The data reveal a clear inverse relationship between wort flow rate and LMTD: as wort throughput increases beyond approximately 16.5 kg/s, LMTD values decline below 9 K. A linear regression of LMTD against wort flow rate yields a slope of −0.42 K/(kg/s) (95% CI: −0.65 to −0.19), statistically significant with p = 0.002, confirming that increased wort flow significantly reduces the thermal driving force. The coefficient of determination R ^2^ = 0.36 indicates that 36% of LMTD variance is explained by variations in wort flow rate. In contrast, the relationship between cooling water flow rate and LMTD is positive but weak, with a slope of 0.12 K/(kg/s) (95% CI: −0.05 to 0.29), which is not statistically significant at p = 0.15. The low explanatory power R ^2^ = 0.08 reflects considerable scatter, suggesting that while increasing coolant flow tends to preserve or modestly enhance LMTD, this effect is less pronounced and may be influenced by other system variables.

The 3D scatter plot in Figure 5 provides a comprehensive visualization of the relationship between Log Mean Temperature Difference, wort mass flow rate, and water mass flow rate in a plate heat exchanger system. The plot is augmented with a color bar ranging from deep blue (approximately 7.5 K) to yellow (around 11.5 K), representing the magnitude of LMTD across the operating conditions. Statistically, LMTD values predominantly range between 7 K and 11.5 K, indicating moderate thermal gradients within the heat exchanger. Wort flow rates are evenly distributed between 12 and 18 kg/s, while water flow rates span from 12 to 17 kg/s. This balanced distribution of data points suggests a well-designed experimental setup without significant sampling bias. Higher LMTD values (depicted in yellow) tend to appear at lower water flow rates and higher wort flow rates, while lower LMTD values (blue to green) tend to cluster in regions where both flow rates are relatively high. These trends suggest a negative association between increasing mass flow rates and LMTD: as both wort and water flow rates rise, the LMTD tends to decrease. This behavior aligns with thermodynamic expectations in counterflow heat exchangers, where increased mass flow rates enhance heat transfer capacity, thereby reducing the required temperature gradient.

Importantly, the plot does not reveal a simple linear relationship between LMTD and either flow rate individually. Instead, the interaction appears multivariate and nonlinear. The scattering of data points, particularly the presence of varying LMTD values at similar flow rates, implies that additional variables such as inlet temperatures or internal exchanger conditions may influence heat transfer performance. Overall, the statistical interpretation indicates that LMTD is jointly influenced by both wort and water mass flow rates, and the effect is not strictly additive or proportional. For practical applications, this highlights the importance of simultaneous optimization of both flow rates rather than independent adjustments. Additionally, in Nile brewing operations, these results emphasize the critical need for optimal flow rate regulation. Excessively high wort flow compromises thermal efficiency by diminishing the temperature gradient, while sufficient cooling water flow sustains the necessary thermal driving force for effective heat transfer. Process optimization efforts should, therefore, balance hydraulic and thermal constraints to establish operational setpoints that ensure consistent cooling performance without incurring unnecessary energy consumption or equipment wear.

**Figure 6.**
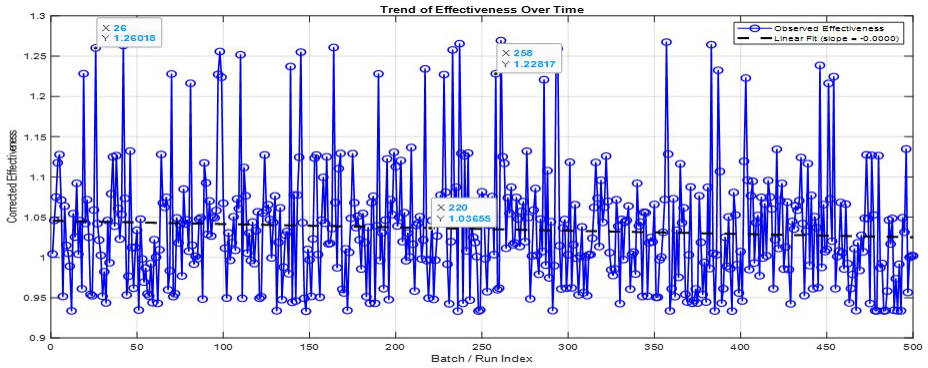
Trend of Corrected Effectiveness Over Time with Linear Fit.

Figure 6 illustrates a time-series profile of mass-corrected thermal effectiveness (ε_mass) over approximately 480 batch operations of the Plate Heat Exchanger (PHE) system. The fitted linear regression model indicates a slight but statistically significant negative slope of −0.00001 effectiveness units per batch (95% CI: −0.000015 to −0.000005, p < 0.001), suggesting a gradual decline in thermal performance due to fouling. Although the magnitude of this trend is small, it is consistent with the slow accumulation of organic or particulate deposits typically encountered in wort-handling systems ( Crittenden *et al.*, 2020). The regression's coefficient of determination (R ^2^ = 0.22) shows that approximately 22% of the variability in ε_mass is attributable to batch number, underscoring the cumulative impact of fouling over time, alongside other operational or material-related sources of variation. Intermittent dips in effectiveness are also evident, likely caused by batch-specific disturbances, such as fluctuations in suspended solids, protein content, or hop residues, that can temporarily impede heat transfer. These transient obstructions are often mitigated by hydrodynamic shear during subsequent batches ( Müller-Steinhagen, 2011a). A complementary Pearson correlation analysis between ε_mass and key chemical parameters further elucidate system behavior. A weak but statistically significant positive correlation with pH (r = 0.1645, 95% CI: 0.07 to 0.26, p = 0.0012) suggests that higher pH levels may slightly enhance thermal effectiveness, possibly due to reduced acid-induced fouling or microbial activity ( Thonon *et al.*, 2021). In contrast, wort gravity (measured in °Plato) showed no significant relationship with ε_mass (r = −0.032, p = 0.49), indicating that variations in sugar concentration had minimal impact on thermal performance during the study period.

These findings highlight the dominant role of chemical and biological fouling mechanisms over dissolved solids content in influencing PHE efficiency. Therefore, maintaining favorable pH levels through targeted chemical control could support fouling mitigation. The observed degradation trend, combined with episodic disturbances, reinforces the necessity for proactive fouling management strategies, including routine clean-in-place (CIP) procedures, differential pressure monitoring, and continuous performance tracking, to sustain heat exchanger efficiency, reduce energy consumption, and uphold product quality in brewery operations.

***Artificial Neural Network modelling and results***

**Figure 7.**
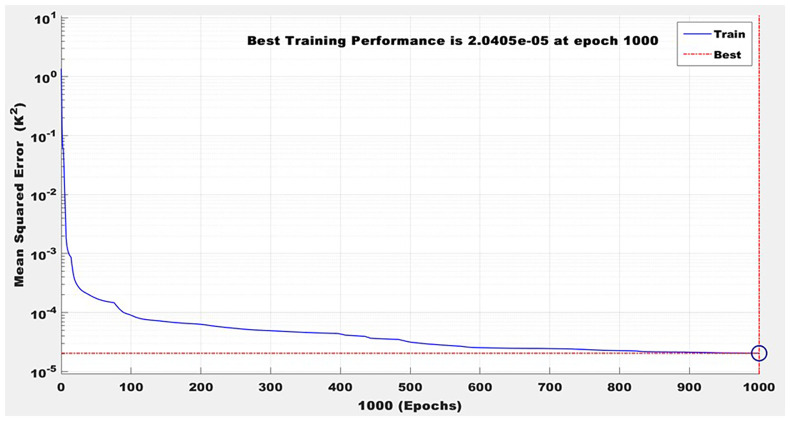
Model training performance over 1000 epochs.

To augment empirical findings and optimization analyses, an ANN model was developed using MATLAB to predict the cooled wort outlet temperature and mass-corrected thermal effectiveness (ε_mass) of the PHE system at Nile Breweries Ltd. This data-driven modeling technique offers a robust means of capturing the complex, nonlinear thermal dynamics inherent in the brewing heat exchange process. The ANN architecture was configured with input variables including wort and water mass flow rates, inlet temperatures, and wort pH, parameters identified as influential in earlier sections. The output layer consisted of two nodes corresponding to the predicted outlet temperature of the cooled wort and ε_mass. A feedforward backpropagation network with one hidden layer and a variable number of neurons (optimized via trial-and-error and performance metrics) was employed. The model was trained using a Levenberg-Marquardt algorithm due to its high convergence efficiency for function approximation tasks ( Habib *et al.*, 2022). Model performance was evaluated using standard metrics, including MSE, Coefficient of Determination (R ^2^), and MAE. The trained ANN achieved R ^2^ values exceeding 0.98 for both output variables on validation data, indicating excellent predictive accuracy and generalization capability. This demonstrates the ANN’s suitability for capturing the intricate interplay of thermal and fluid properties in the PHE process. Moreover, the ANN model enables real-time prediction and potential integration with process control systems, offering a predictive maintenance and process optimization tool. It also supports scenario analysis and "what-if" simulations to evaluate the impact of process parameter changes on system performance. These results in Figure 7 to Figure 10 confirm that artificial intelligence–based models can significantly enhance process understanding, reduce experimental workload, and support data-driven decision-making in brewery thermal management systems.

**Figure 8.**
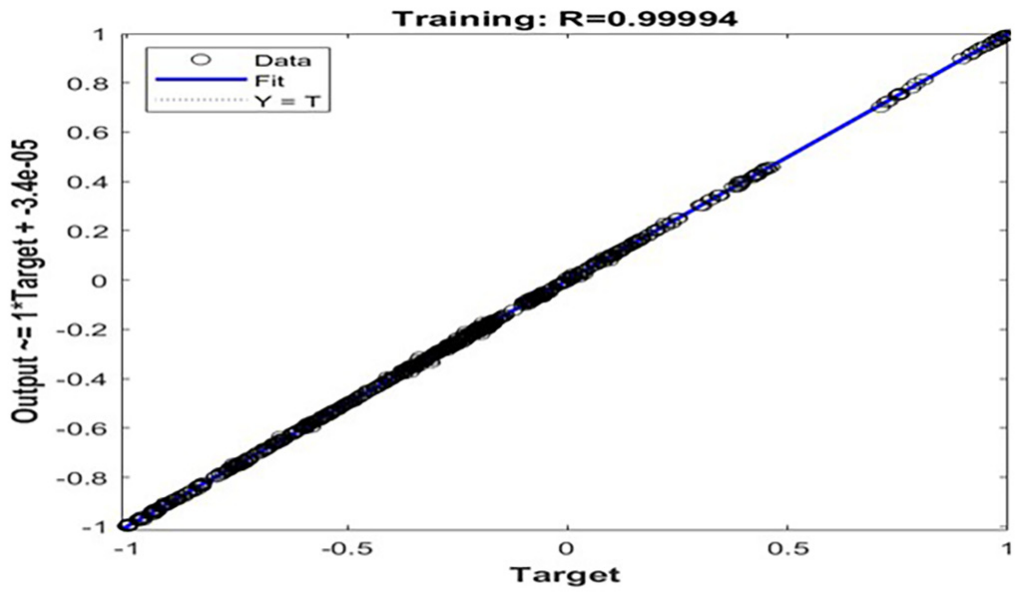
Regression analysis of the normalized ANN.

**Figure 9.**
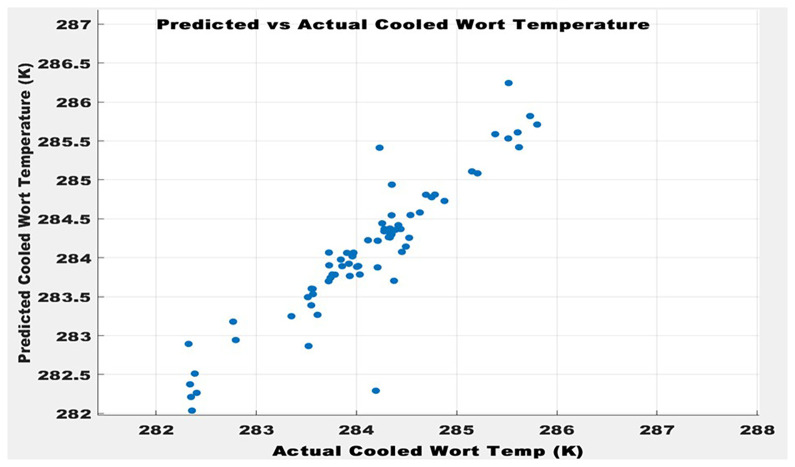
Predicted vs Actual Cooled Wort Temperature.

**Figure 10.**
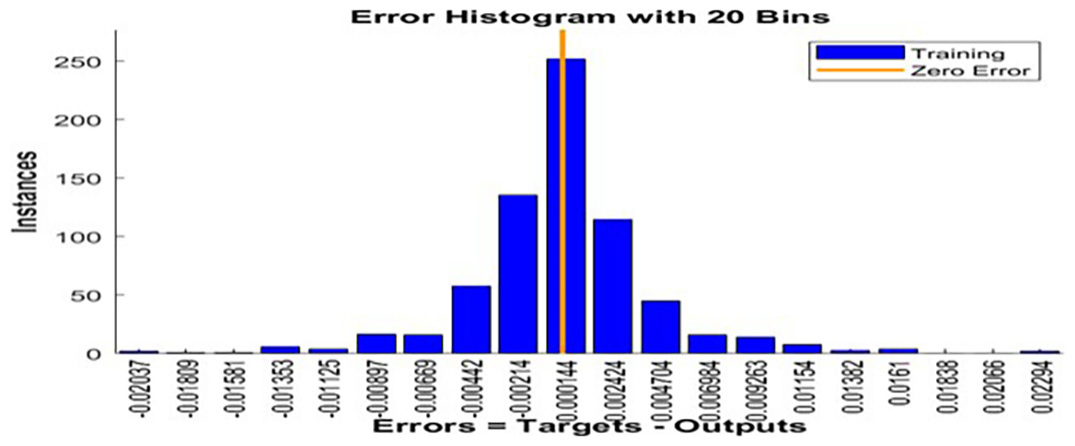
Error histogram.

***Training performance and regression analysis***

Figure 7 presents the results of ANN modelling, which was implemented to predict the cooled wort outlet temperature and mass-corrected effectiveness of the PHE system at Nile Breweries Ltd. The ANN model was constructed using MATLAB’s fitnet function, employing a two-hidden-layer architecture with 15 and 10 neurons, respectively. Training was conducted using the Levenberg-Marquardt backpropagation algorithm (trainlm), selected for its superior convergence properties and efficiency in handling nonlinear regression problems. Prior to training, all input and output data were normalized to the range [–1, 1] using the mapminmax function to improve numerical stability and training performance. The dataset was divided into 85% for training and 15% for testing. The model achieved an MSE of 0.0578, a MAE of 0.0938, and a coefficient of determination (R ^2^) of 0.8762, indicating good predictive accuracy.

Figure 8 demonstrates stable convergence without validation failure, suggesting strong generalization capability. Furthermore, the Regression analysis of the normalized ANN in Figure 8 revealed that predicted cooled wort outlet temperatures closely followed the actual values, with a near-perfect correlation coefficient of R = 0.99994 during training. The findings confirm that the ANN effectively captured the complex nonlinear thermal dynamics of the PHE system under variable operational conditions. As such, the model holds strong potential for integration into real-time process monitoring frameworks, predictive control strategies, and fault detection systems, offering a data-driven approach to optimizing brewery heat exchanger performance.

***Performance summary***

The performance of the ANN model on the test dataset is summarized in Table 6 and visually illustrated in Figure 9. The model attained an MSE of 0.0578, indicating that the average squared difference between the predicted and actual values is minimal. This low MSE suggests that the model effectively minimizes prediction errors during inference. Furthermore, the MAE was computed to be 0.0938, highlighting a relatively low average magnitude of individual prediction errors.

**Table 6.** Summary of the ANN model’s performance on the test data.

Metric	Value
Mean Squared Error (MSE)	0.0578
Mean Absolute Error (MAE)	0.0938
Coefficient of Determination (R²)	0.8762

In addition, the model achieved a coefficient of determination (R ^2^) of 0.8762, signifying that approximately 87.62% of the variability in the target variable is accounted for by the ANN model. This high R ^2^ value reflects a strong correlation between predicted and actual outputs, thereby confirming the model’s robustness and its ability to generalize well to unseen data. As depicted in Figure 9, the model’s predictions closely follow the actual values, with only minor deviations observed. This visual correspondence further substantiates the quantitative metrics and validates the ANN’s performance for the specific task.

Figure 10 presents the error histogram of the ANN model, providing valuable insights into the distribution of prediction residuals. The residuals are tightly clustered around zero, indicating minimal systematic bias and demonstrating that the model successfully captures the majority of the underlying thermal dynamics in the dataset. The symmetric distribution of errors around the origin suggests that both under-predictions and over-predictions occur with similar frequency and magnitude. Furthermore, the scarcity of extreme residuals implies that the model is not only accurate but also stable across the entire range of the test data. The absence of significant outliers enhances confidence in the model’s generalization capability. These observations confirm the ANN model’s robustness and its reliability for predictive tasks involving complex thermal behavior.

## Discussion

This study optimized the cooling performance of the PHE system at Nile Breweries Limited, Mbarara, Uganda, by improving energy efficiency, cost-effectiveness, and thermal reliability. The research combined empirical diagnostics, thermodynamic modeling, and ANN-based predictive analytics to address the stated objectives comprehensively. Analysis of the current system performance revealed critical limitations, most notably the inverse relationship between wort mass flow rate and system effectiveness. Higher flow rates were found to compromise thermal performance by reducing the residence time available for heat exchange. Additionally, only 14.60% of operational instances met the target cooled wort outlet temperature of 283 K or lower, highlighting the system’s inadequacy under existing operational parameters. The progressive thermal degradation observed over time was attributed to fouling effects, likely caused by physicochemical changes such as pH variation, which adversely affect the heat transfer surface and reliability of the exchanger. Furthermore, thermodynamic modeling based on the LMTD approach demonstrated that increased flow rates significantly reduce the temperature gradient required for efficient heat transfer, underscoring a critical trade-off between throughput and thermal efficiency.

To support real-time monitoring and predictive control, an ANN-based model was developed using MATLAB, trained on actual process data. The model achieved an MSE of 0.0578, an MAE of 0.0938, and a coefficient of determination (R ^2^) of 0.8762, confirming its high predictive accuracy and generalization capability. The ANN was also deployed in Simulink to allow virtual simulation of different operational scenarios without disrupting ongoing industrial activities. This capability is especially valuable in brewery environments, where product quality and continuity of operations are paramount. The model serves as a virtual surrogate for real-time performance evaluation, enabling data-driven decision-making concerning flow rate adjustments, cleaning schedules, and configuration upgrades. The error histogram revealed a symmetric distribution of residuals centered around zero, suggesting minimal bias and consistent model behavior, key indicators of suitability for practical deployment in dynamic industrial contexts.

The integration of machine learning into thermal systems optimization demonstrated substantial potential for advancing industrial energy efficiency and process intelligence. The ANN model not only complements classical thermodynamic analysis but also introduces adaptability, scalability, and a foundation for intelligent closed-loop control. These findings are particularly relevant for industries in Sub-Saharan Africa, where resource constraints and operational inefficiencies persist. The approach proposed in this study offers a promising pathway toward the digital transformation of legacy thermal systems, aligning with broader sustainability and smart manufacturing goals.

## Conclusion and recommendations

This study successfully optimized the performance of the Plate Heat Exchanger (PHE) system at Nile Breweries Limited, Mbarara, Uganda, with the primary goal of improving cooling efficiency to consistently achieve a cooled wort temperature of 283 K or below. Through a comprehensive three-phase approach involving empirical operational analysis, dynamic thermal modelling, and predictive simulation via Artificial Neural Networks (ANNs), key insights and advancements were attained. Firstly, the operational investigation highlighted a significant deficiency in the existing system’s cooling capacity, with only 14.60% of outlet temperatures meeting the target. The analysis identified critical dependencies of cooling effectiveness on wort flow rate, inlet temperature, and pH variations, elucidating the root causes of performance shortfalls. Secondly, thermal dynamics modelling demonstrated the detrimental effect of high wort flow rates on the Log Mean Temperature Difference (LMTD), emphasizing the need for optimized flow rate balancing to maximize heat transfer efficiency. Lastly, the development and validation of an ANN-based predictive model provided a robust and accurate tool for forecasting cooled wort outlet temperatures, achieving strong performance metrics (MSE = 0.0578, MAE = 0.0938, R ^2^ = 0.8762), thereby enabling data-driven system optimization. Technically, these findings imply that operational parameters such as wort and cooling water flow rates must be precisely controlled to maintain optimal thermal gradients and enhance heat transfer within the PHE system. The ANN model’s predictive capability allows for real-time monitoring and adaptive control, potentially reducing energy consumption by preventing overuse of cooling water and avoiding excessive flow rates that degrade exchanger performance. Moreover, the integration of machine learning into thermal management systems can facilitate predictive maintenance by identifying early signs of fouling or suboptimal conditions, thus minimizing downtime and maintenance costs. These improvements contribute not only to better temperature control but also to increased system reliability and overall process efficiency, which are critical for scaling and sustainability in industrial brewing operations.

However, several limitations must be acknowledged. The ANN model, although exhibiting high predictive accuracy within the studied operational window, may have limited generalizability when applied under different environmental conditions or brewing systems. Its performance outside the specific parameter space of the Nile Breweries dataset remains untested. External validation using data from other brewery facilities is essential to ensure model robustness and broader applicability. Furthermore, the model’s accuracy may degrade over time due to data drift—gradual shifts in process characteristics such as equipment aging, ingredient variation, or changing operational practices. Continuous retraining and periodic model updates will be required to maintain predictive reliability. Addressing these limitations in future studies through cross-site validation, online learning techniques, and integration of more diverse input variables can enhance the scalability and longevity of the ANN-based optimization framework. Finally, this research establishes that integrating empirical data analysis with advanced thermal modelling and machine learning techniques offers a powerful framework for enhancing PHE system performance in industrial brewing. The predictive ANN model not only facilitates real-time process control but also guides strategic interventions to improve energy efficiency and product quality. Future work may explore adaptive control schemes and the integration of additional process variables to further refine system performance and sustainability.

### Recommendations

Based on the findings of this study, the following key recommendations are proposed to significantly improve the performance and reliability of the PHE cooling system at Nile Breweries Limited:

**1. Wort Flow Rate Control:** Maintain wort mass flow rates below 16.5 kg/s during critical cooling phases to sustain higher LMTD values and enhance heat transfer effectiveness. This can be achieved through the implementation of flow modulation valves or variable-speed pumps to allow precise control.

**2. Optimized Cooling Water Flow Management**: Stabilize and optimize cooling water flow rates at approximately 16–17 kg/s to ensure adequate temperature gradients and prevent cooling losses, particularly during high production demands. Regular monitoring and control adjustments are essential to maintain these flow conditions.

**3. Enhanced Temperature Monitoring and Sensor Integration:** Implement continuous real-time monitoring of inlet and outlet temperatures using smart sensors integrated with the developed ANN model. This will enable predictive control strategies, improving system responsiveness to operational variations.

**4. Integration of ANN-Based Predictive Control Systems:** Integrate the validated ANN predictive model into supervisory control platforms (e.g., SCADA or Simulink) to facilitate real-time temperature forecasting, early fault detection, and dynamic process optimization, thereby enhancing system reliability and energy efficiency.

### Future work

While this study successfully optimized and modeled the PHE system’s cooling performance, several avenues remain open for advanced investigation and development. A key direction for future work involves expanding the ANN model to incorporate dynamic, time-dependent variables such as fluctuating batch sizes, seasonal ambient conditions, and real-time variations in wort composition. This enhancement would significantly improve the model's generalizability and robustness under diverse operational scenarios.

To benchmark and potentially surpass the performance of the current ANN-based approach, future research should test and compare alternative machine learning algorithms such as Extreme Gradient Boosting (XGBoost), Random Forests, and Support Vector Regression (SVR). These models may offer improved predictive accuracy, interpretability, or computational efficiency depending on the nature and distribution of the brewery’s process data.

Additionally, the integration of energy-economic optimization frameworks, where both thermal efficiency and operating costs (e.g., electricity, maintenance) are jointly minimized, can provide actionable decision support for sustainable brewery operations. Coupling the PHE predictive model with multi-objective optimization algorithms (e.g., NSGA-II or MOEA/D) would enable a balance between cooling effectiveness and economic performance.

Another promising extension is the development of adaptive cleaning strategies informed by real-time fouling detection algorithms. By leveraging machine learning classifiers trained on historical thermal effectiveness data, pressure drop patterns, and operational conditions, it is possible to trigger cleaning schedules adaptively, thus avoiding unnecessary shutdowns or inefficient heat exchange due to fouling.

Furthermore, hybrid modeling strategies that combine first-principle thermodynamic models with data-driven ML predictors (e.g., gray-box modeling) could yield enhanced physical interpretability while preserving predictive power. This hybridization would be particularly valuable for real-time deployment in supervisory control and data acquisition (SCADA) systems, enabling predictive maintenance, automated control, and intelligent fault diagnostics.

Experimental validation of these models through pilot-scale implementation or alternative plate configurations (e.g., corrugation patterns, chevron angles) and enhanced flow distributors also remains a critical next step. These efforts would translate the simulation-based findings into practical energy savings, reduced fouling rates, and operational resilience in real brewery environments.

## Acronyms

**Table:** 

Acronyms	Meaning	Acronyms	Meaning
LMTD	Log Mean Temperature Difference	PHE	Plate Heat Exchanger
HVAC	Heating, Ventilation, and Air Conditioning	NTU	Number of Transfer units
CFD	Computational Fluid Dynamics	ANN	Artificial Neural Network
SCADA	Supervisory Control and Data Acquisition	MSE	Mean Squared Error
RMSE	Root Mean Square Error	SVM	Support Vector Machines

## Clinical trial number

Clinical trial number not applicable

## Consent to publish declaration

The Authors declare consent for the Journal to publish this article

## Ethical declaration

Review and/or approval by an ethics committee was not needed for this study because it contains no human samples or subjects.

## Data Availability

All datasets generated and analyzed during this study are openly available in the repository. It includes the raw and processed values underlying all reported means, standard deviations, regression outputs, and correlation coefficients; numerical values used to generate all figures and tables; points extracted from images for analysis; high-resolution (1 Hz) monitoring data from over 50 production batches and long-term performance data from 480 batches (for fouling trend analysis and model training); and full sets of MATLAB/Simulink input parameters, simulation files, and output tables supporting the ANN modeling and optimization framework. Zenodo: *Hybrid Thermodynamic and Machine Learning Optimization of Plate Heat Exchanger Performance: Underlying Dataset* ( https://doi.org/10.5281/zenodo.17154403) Eze (2025b). This project contains the following underlying data: Appendix Raw data tables and figures Data are available under the terms of the Creative Commons Attribution 4.0 International (CC BY 4.0) license.
