# Supplementary material for: Hybrid Thermodynamic and Machine Learning Optimization of Plate Heat Exchanger Performance for Brewery Cooling Systems in Uganda
Source: Open Res Afr. 2025 Nov 3;8:24. [Version 1] doi: 10.12688/openresafrica.16068.1 (PMC12963833; doi:10.12688/openresafrica.16068.1)
Supplement: Peer review history

# Reviewer report: Reviewer response for version 1

**DOI:** 10.21956/openresafrica.17243.r33798

**Published:** 2026-03-05

**Authors:** Ashan Induranga

**Affiliations:** 1 Department of Engineering Technology, Sabaragamuwa University of Sri Lanka, Belihuloya, Sri Lanka

1. Some of the abbreviations are not mentioned in the first place. (Ex: LMTD)
2. The authors have prepared the manuscript in a well-organized manner, which should be appreciated.
3. The authors have explained the mathematical significance relevant to graphs well.
4. It is recommended to provide a diagram; photograph of the Data Acquisition System with the Data Acquisition Device used in the study.

Is the work clearly and accurately presented and does it cite the current literature?

Yes

If applicable, is the statistical analysis and its interpretation appropriate?

Yes

Are all the source data underlying the results available to ensure full reproducibility?

Yes

Is the study design appropriate and is the work technically sound?

Yes

Are the conclusions drawn adequately supported by the results?

Yes

Are sufficient details of methods and analysis provided to allow replication by others?

Yes

Reviewer Expertise:

Thermal and Di-Electric Applications of Nanofluids, Data Acquisition Systems, Control Systems

I confirm that I have read this submission and believe that I have an appropriate level of expertise to confirm that it is of an acceptable scientific standard.

# Reviewer report: Reviewer response for version 1

**DOI:** 10.21956/openresafrica.17243.r33007

**Published:** 2025-11-25

**Authors:** Ibrahim Mahariq

**Affiliations:** 1 Al-Quds University, Jerusalem, Palestinian Territory

The authors have to revise the manuscript and address the below comments: 

- The methodology section is comprehensive but somewhat dense; the flow between empirical data processing, theoretical modeling, and ANN implementation could be made clearer by adding a short roadmap paragraph or schematic summarizing how each methodological component links to each research objective. This will help readers follow the reasoning and workflow more easily.
- Please ensure that all figures include units on their axes where applicable (e.g., Figures 5 and 9) to improve interpretability.
- Some regression equations and symbols appear multiple times in slightly different forms; harmonizing notation and ensuring consistency across all formulae would improve readability.
- A few references in the introduction and methods sections appear repeated (e.g., the same author group cited twice in the same year); please check for duplication and formatting consistency according to journal style.
- Consider expanding the caption text for Figures 7–10 to briefly explain their significance rather than only describing what they display (e.g., explicitly noting how they demonstrate model validity).
- the authors have to enrich the introduction section by citing the following strongly relavent articles: a1- Thermal frictional analysis of a novel vibrating cylindrical turbulator in double tube heat exchangers for engine cooling  a2- Enhancing the heat transfer efficiency of an engine heat exchanger through the utilization of an innovative conical braided wire turbulator  a3- Thermal enhancement of a heat exchanger of engine via U-cut and V-cut zigzag strip turbulator combined with bubble injection method  a4- Performance analysis of spiral heat exchanger based on E-RPN and BWM multi-criteria decision algorithm-application of combined heat and power systems

Is the work clearly and accurately presented and does it cite the current literature?

Yes

If applicable, is the statistical analysis and its interpretation appropriate?

Yes

Are all the source data underlying the results available to ensure full reproducibility?

Yes

Is the study design appropriate and is the work technically sound?

Yes

Are the conclusions drawn adequately supported by the results?

Yes

Are sufficient details of methods and analysis provided to allow replication by others?

Yes

Reviewer Expertise:

computational fluid dynamics

I confirm that I have read this submission and believe that I have an appropriate level of expertise to confirm that it is of an acceptable scientific standard.
